# Supplementary material for: Learning alters salience and proactive attentional priority
Source: Commun Psychol. 2026 Feb 17;4:57. doi: 10.1038/s44271-026-00411-0 (PMC13021526; doi:10.1038/s44271-026-00411-0)
Supplement: Supplementary file 2 — Supplementary material [file 44271_2026_411_MOESM2_ESM.pdf]

Supplementary Figures

From the article “Learning Alters Salience and Proactive Attentional Priority” published in Communications Psychology (2026)

	No Dist.	*HP*	*1*	*2*	*3*	*4*
No Dist.		***	***	***	***	***
HP	***		***	***	***	***
1	***	***		***	***	***
2	***	***	***		ns	ns
3	***	***	***	ns		ns
4	***	***	***	ns	ns	

Supplementary Table 1 *– Distractor Location RT Differences as a function of distance from HP location in Experiments 1A and 1B.. Shown is the matrix of pairwise comparisons between distractor as a function of the distractors current distance away from HP distractor location. This table is an accompaniment to Figure 1D. cells labeled 1-4 represent distance away from the HP location (e.g. 2 represents two locations in space away from the HP location). clockwise and counterclockwise distance are collapsed in this figure, but separated in Supplementary Figure 2. Each cell represents the outcome of a two-tailed repeated measures t-test. * = p < 0.05; ** = p < 0.01; *** = p < 0.001.*

	HP	*hpt*	*top*	*lpt*	*LP*	*lpb*	*Bottom*	*hpb*
HP		***<br>***	***<br>***	***<br>***	***<br>***	***<br>***	***<br>***	***<br>***
hpt	***<br>***		**<br>**	*<br>*	*<br>*	ns<br>ns	ns<br>ns	**<br>ns
top	***<br>***	**<br>**		ns<br>ns	ns<br>ns	ns<br>*	ns<br>ns	***<br>***
lpt	***<br>***	*<br>*	ns<br>ns		ns<br>ns	ns<br>*	ns<br>ns	***<br>**
LP	***<br>***	*<br>*	ns<br>ns	ns<br>ns		ns<br>*	ns<br>ns	***<br>ns
lpb	***<br>***	ns<br>ns	ns<br>*	ns<br>*	ns<br>*		ns<br>*	***<br>**
Bottom	***<br>***	ns<br>ns	ns<br>ns	ns<br>ns	ns<br>ns	ns<br>*		***<br>**
hpb	***<br>***	**<br>ns	***<br>***	***<br>**	***<br>**	***<br>ns	***<br>**	

Supplementary Table 2 *– Distractor Location RT Differences. Shown is the matrix of pairwise comparisons between distractor locations separated for the eight locations in space and arranged relative to the HP location. Significance stars for the combined data of Experiments 1A and 1B are denoted in black, while those of Experiment 2 are denoted below in green. LP represents the horizontal location on the opposite side of the screen from the HP location. Labels correspond to those used in Figure 1E. HP = the horizontally placed high-probability distractor location; hpt = the upper diagonal location in the same hemifield as the HP location; top = top of the screen; lpt = upper diagonal location in the opposite hemifield as the HP location; LP = the horizontally placed low-probability distractor location on the opposite side of the screen from the HP location;; lpb = the lower diagonal location in the opposite hemifield as the HP location; bottom = bottom of the screen; hpb = lower diagonal location in the same hemifield as the HP location. Each cell represents the outcome of a two-tailed repeated measures t-test. * = p < 0.05; ** = p < 0.01; *** = p < 0.001.*

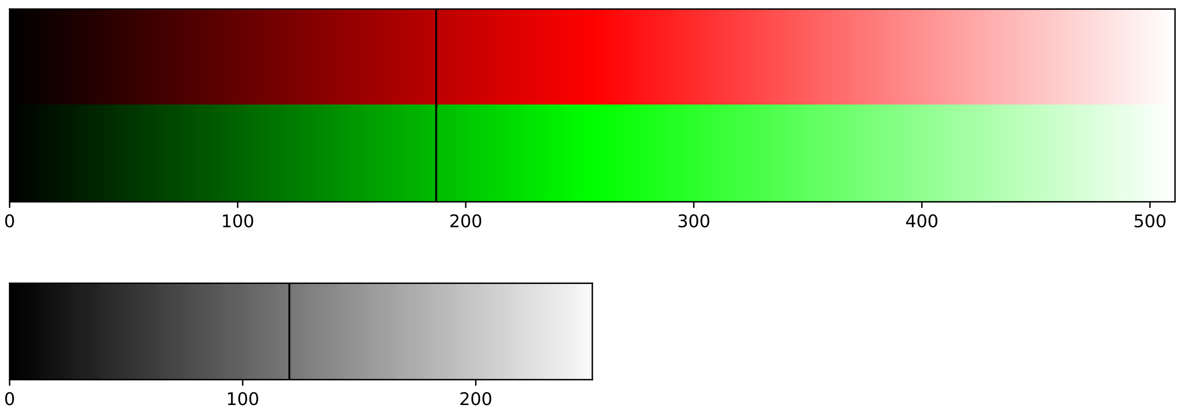

Supplementary Figure 1 *- color spectra used for probe tests in Experiments 1 (top) and 2 (bottom). Hexadecimal color names were used starting at black (#000000) and ending at white (#FFFFFF) with red, green or grey increasing their value monotonically from left to right. The black horizontal line represents the color of the neutral probe on every trial (located at hexadecimal values #BB0000, #00BB00 and #787878 for red, green and grey respectively. This corresponded to position 187 in the color spectra or position 120 in the greyscale spectra). The probe color was slightly offset from the center of the spectra in both cases as it was found that darker tones were easier to distinguish from nearby values than lighter tones in pilot studies. Grey probes used position 100 as the standard color instead of position 187 because the grey stimulus space was exactly half as long as the colored stimulus space, meaning position 187 would already be relatively close to the maximum white value..*

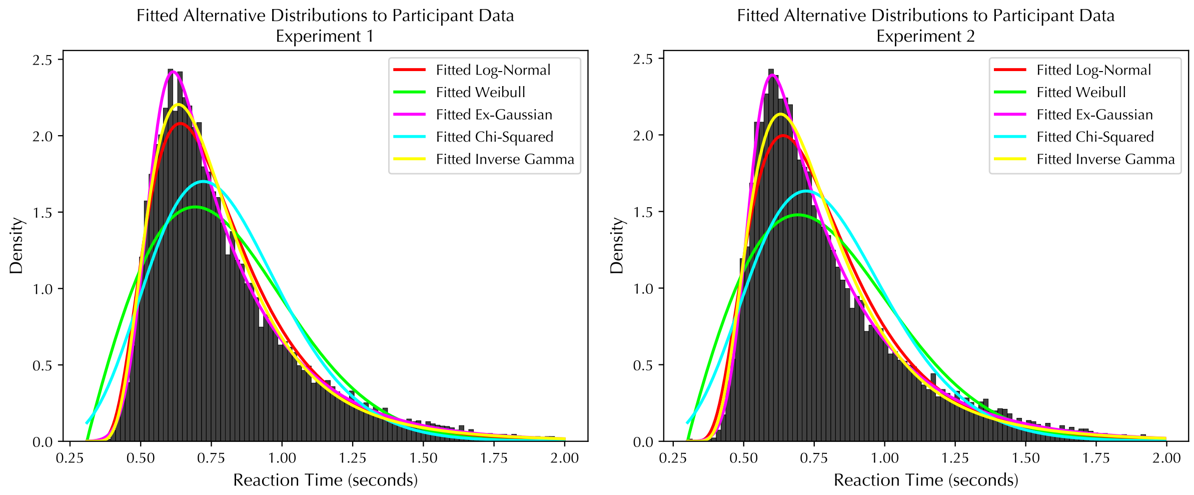

Supplementary Figure 2 *– Candidate curves fit to no-distractor trials to determine the underlying distribution of response times fitted to data from Experiments 1 and 2. Different curves are represented in separate colors coded in the figure legend. Probability density functions were calculated for each curve and used to find the log-likelihood of each curve given the underlying data distribution. Log-likelihoods were then used to calculate AIC and BIC scores from which the log-normal distribution had the lowest value in both measures, thus representing the best fit to the data.*
